# Supplementary figures and images for: Individual small in‐stream barriers contribute little to strong local population genetic structure five strictly aquatic macroinvertebrate taxa
Source: Ecol Evol. 2022 Apr 13;12(4):e8807. doi: 10.1002/ece3.8807 (PMC9006233; doi:10.1002/ece3.8807)

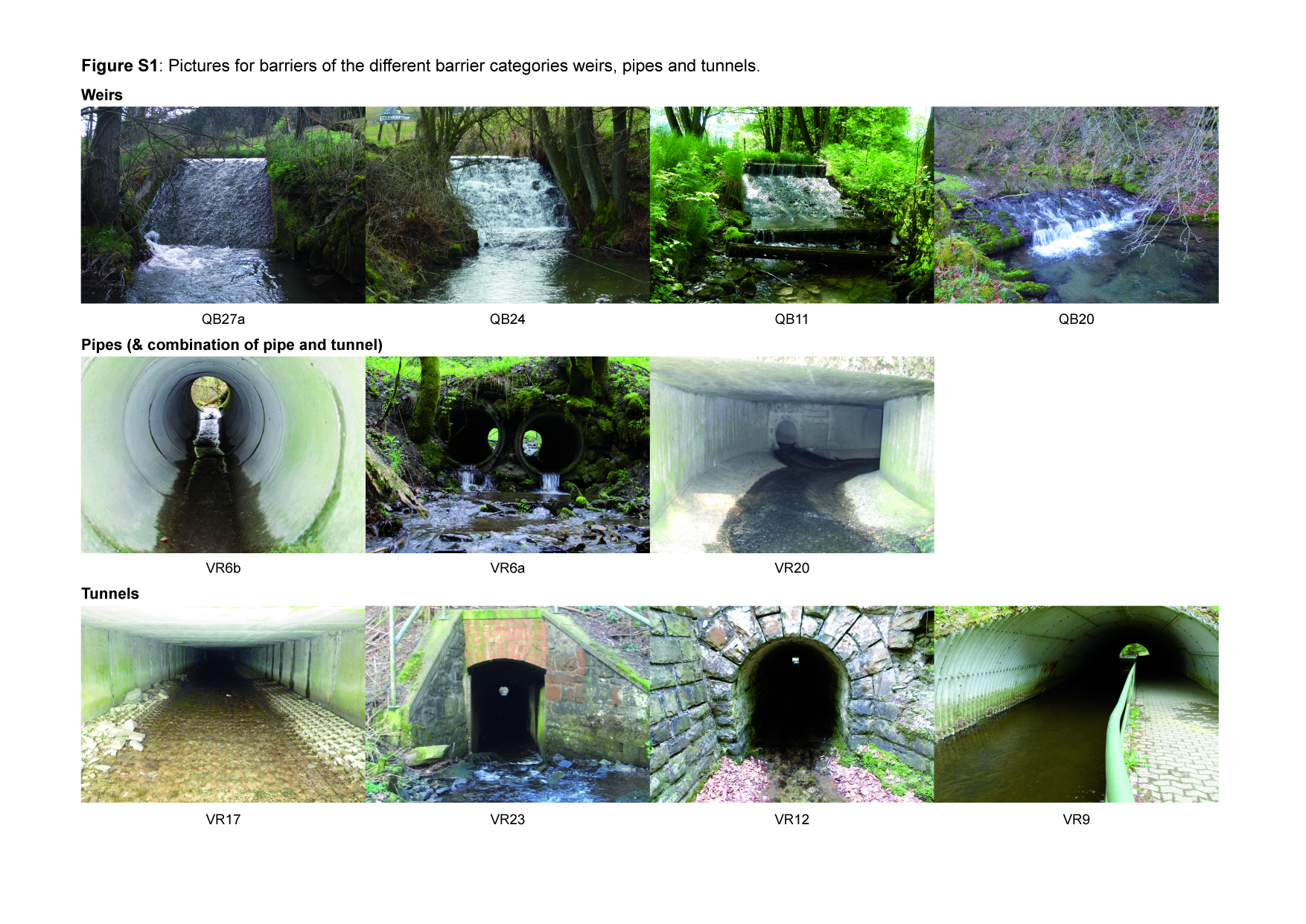

Supplement: Supplementary file 1 — Figure S1 [file ECE3-12-e8807-s004.tif]
