# Supplementary material for: Individual small in‐stream barriers contribute little to strong local population genetic structure five strictly aquatic macroinvertebrate taxa
Source: Ecol Evol. 2022 Apr 13;12(4):e8807. doi: 10.1002/ece3.8807 (PMC9006233; doi:10.1002/ece3.8807)
Supplement: Supplementary file 3 — Figure S3 [file ECE3-12-e8807-s017.pdf]

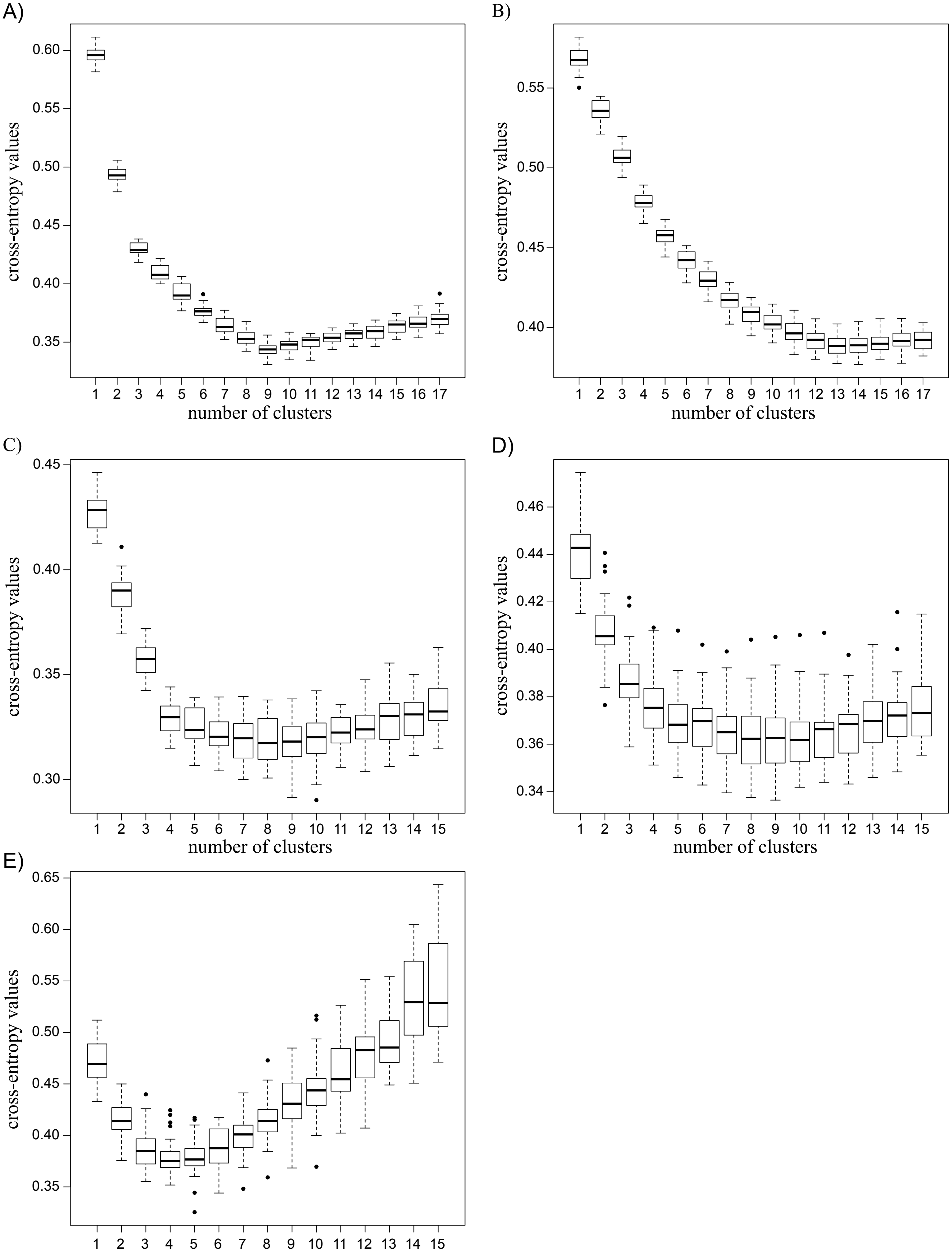

Figure S3. Standard boxplots of cross-entropy values (30 repeats) of sNMF analysis for final ddRAD datasets for the different taxa. A) *G. fossarum*, B) *D. gonocephala,* C-E) *A. fluviatilis* I, II and III.
