# Supplementary material for: Individual small in‐stream barriers contribute little to strong local population genetic structure five strictly aquatic macroinvertebrate taxa
Source: Ecol Evol. 2022 Apr 13;12(4):e8807. doi: 10.1002/ece3.8807 (PMC9006233; doi:10.1002/ece3.8807)
Supplement: Supplementary file 4 — Figure S4 [file ECE3-12-e8807-s005.pdf]

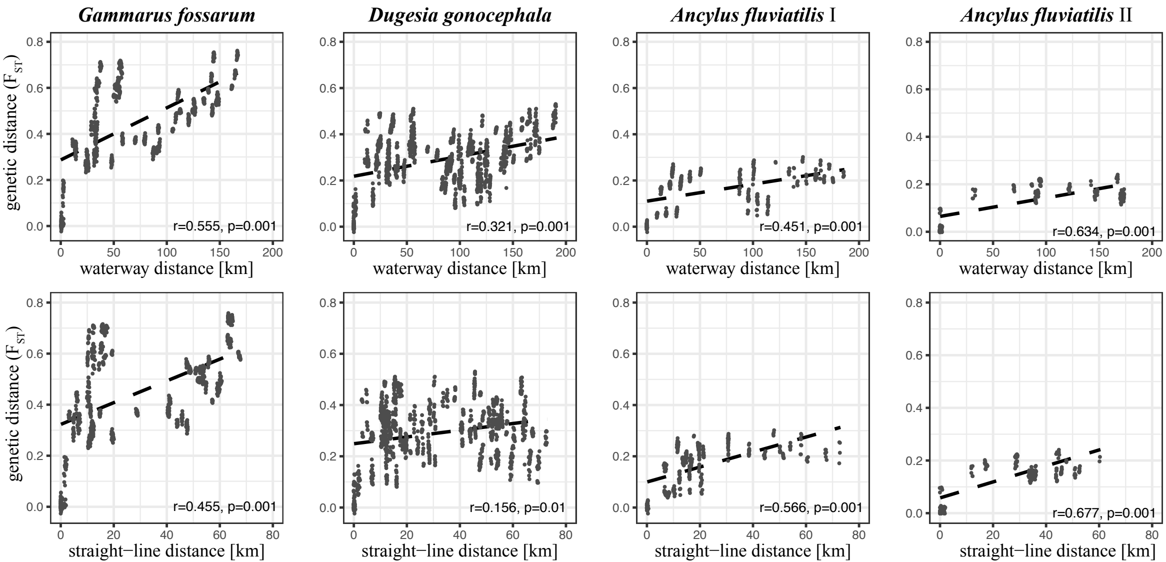

Figure S4. Correlation between pairwise genetic distance (F_{ST}) among single sampling sites and geographic distances (first row waterway distance; second row straight-line distance) for *G. fossarum*, *D. gonocephala*, *A. fluviatilis* I and *A. fluviatilis* II (different columns).
