# Supplementary material for: Individual small in‐stream barriers contribute little to strong local population genetic structure five strictly aquatic macroinvertebrate taxa
Source: Ecol Evol. 2022 Apr 13;12(4):e8807. doi: 10.1002/ece3.8807 (PMC9006233; doi:10.1002/ece3.8807)
Supplement: Supplementary file 5 — Figure S5 [file ECE3-12-e8807-s015.pdf]

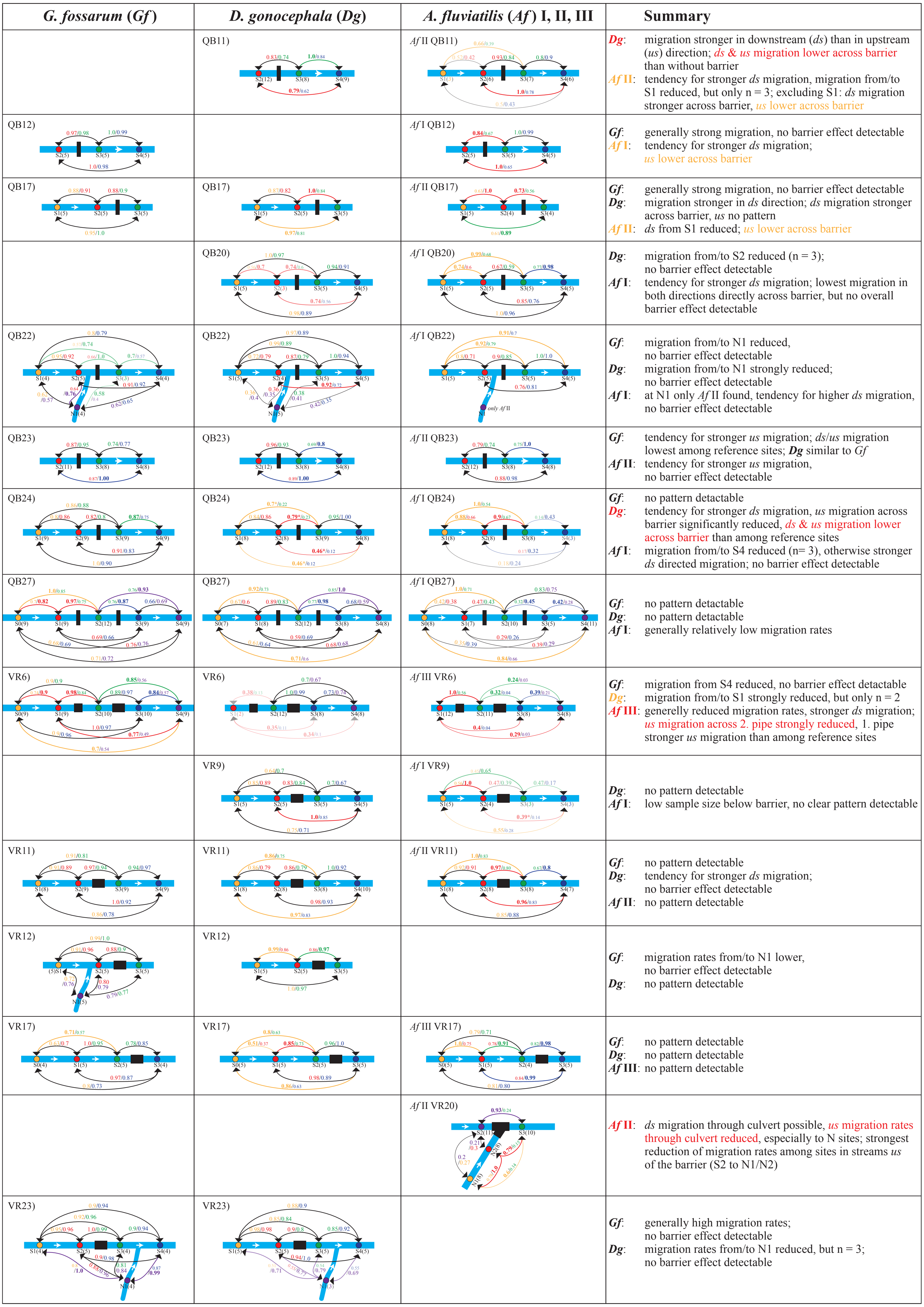

Figure S5. Vizualisation of asymmetric migration rates among all single sampling site calculated seperately for each barrier. Rates are colored according to source site and arrows are colored if rates differed more than 0.15 and then according to the direction of the higher rate. Significant asymmetry is indicated by an asterisk. If sample size was ≤ 3, rates are shown in lighter color.
