# Supplementary material for: Individual small in‐stream barriers contribute little to strong local population genetic structure five strictly aquatic macroinvertebrate taxa
Source: Ecol Evol. 2022 Apr 13;12(4):e8807. doi: 10.1002/ece3.8807 (PMC9006233; doi:10.1002/ece3.8807)

Appendix S3: Results of the Linear mixed models and plots showing the distribution of F_{ST} values in the different categories. For model 1 categories of fixed effects are if a barrier was present (barrieryes) or not present (barrierno) between populations; for model 2 barriers were subdivided into barrier type, i.e. weir (QB), pipes (PI), and tunnels (VR).

A) Gammarus fossarum

Model 1

Linear mixed model fit by REML. t-tests use Satterthwaite's method ['lmerModLmerTest']
Formula: Fst ~ barrier + (1 | site)
Data: Gf

REML criterion at convergence: -350.6
Scaled residuals:

Min	1Q	Median	3Q	Max
-2.3498	-0.4975	-0.1185	0.3279	4.1682

Random effects:

Groups	Name	Variance	Std.Dev.
site	(Intercept)	1.20E-05	0.003462
Residual		5.25E-05	0.007248

Number of obs: 54, groups: site, 13

Fixed effects:

	Estimate	Std. Error	df	t value	Pr(>|t|)
(Intercept)	0.005447	0.001942	35.5473	2.804	0.00812
barrieryes	-0.005365	0.002073	41.950435	-2.589	0.01318

Correlation of Fixed Effects:

	(Intr)
barrieryes	-0.695

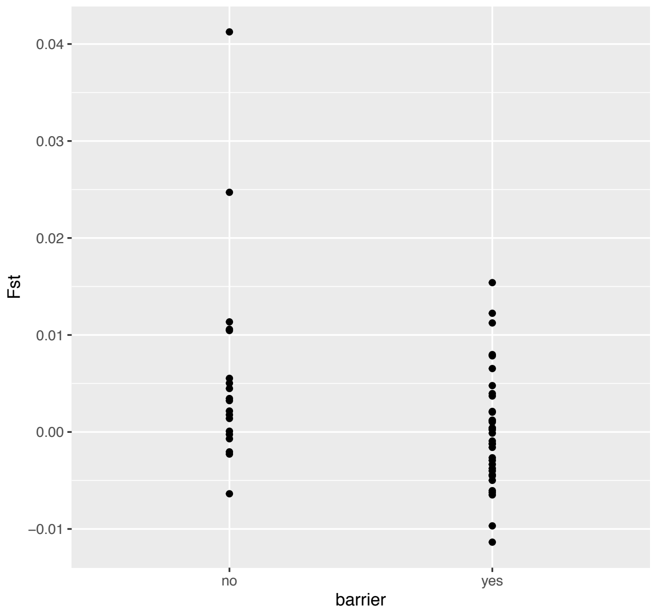

Model 2:

Linear mixed model fit by REML. t-tests use Satterthwaite's method ['lmerModLmerTest']
Formula: Fst ~ QB_VR + (1 | site)
Data: Gf

REML criterion at convergence: -335.1

Scaled residuals:

Min	1Q	Median	3Q	Max
-2.2227	-0.4528	-0.1817	0.3692	4.0321

Random effects:

Groups	Name	Variance	Std.Dev.
site	(Intercept)	1.02E-05	0.003197
Residual		5.25E-05	0.007242

Number of obs: 54, groups: site, 13

Fixed	effects:				
	Estimate	Std. Error	df	t value	Pr(>|t|)
(Intercept)	0.007317	0.002223	34.453138	3.291	0.00231
QB_VRPI	-0.004430	0.004566	45.357405	-0.970	0.33709
QB_VRQB	-0.007590	0.002805	39.063663	-2.706	0.01004
QB_VRVR	-0.007312	0.002914	48.530008	-2.510	0.01547

Correlation of Fixed Effects:

	(Intr)	QB_VRP	QB_VRQ
QB_VRPI	-0.407		
QB_VRQB	-0.724	0.295	
QB_VRVR	-0.548	0.223	0.397

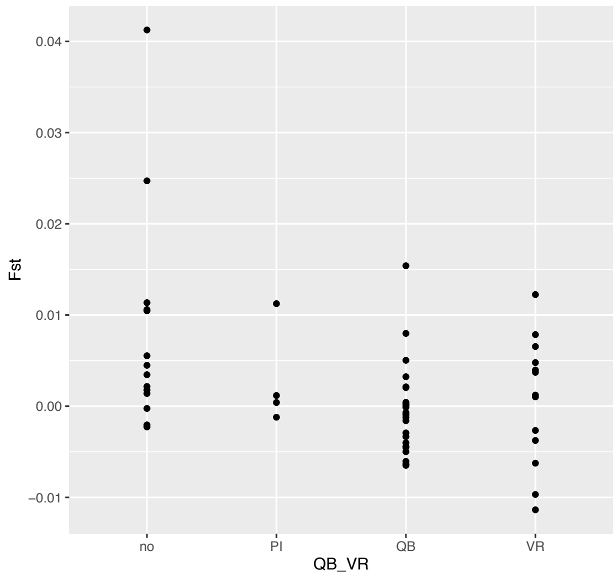

B) Dugesia gonocephala

Model 1

Linear mixed model fit by REML. t-tests use Satterthwaite's method ['lmerModLmerTest']
Formula: Fst ~ barrier + (1 | site)
Data: Dg

REML criterion at convergence: -283.6

Scaled residuals:

Min	1Q	Median	3Q	Max
-2.1552	-0.2949	-0.0982	0.169	3.659

Random effects:

Groups	Name	Variance	Std.Dev.
site	(Intercept)	0.0001902	0.01379
Residual		0.0004301	0.02074

Number of obs: 64, groups: site, 15

Fixed effects:

	Estimate	Std. Error	df	t value	Pr(>|t|)
(Intercept)	0.005583	0.005794	35.565882	0.964	0.342
barrieryes	0.005609	0.005486	51.171561	1.022	0.311

Correlation of Fixed Effects:

	(Intr)
barrieryes	-0.629

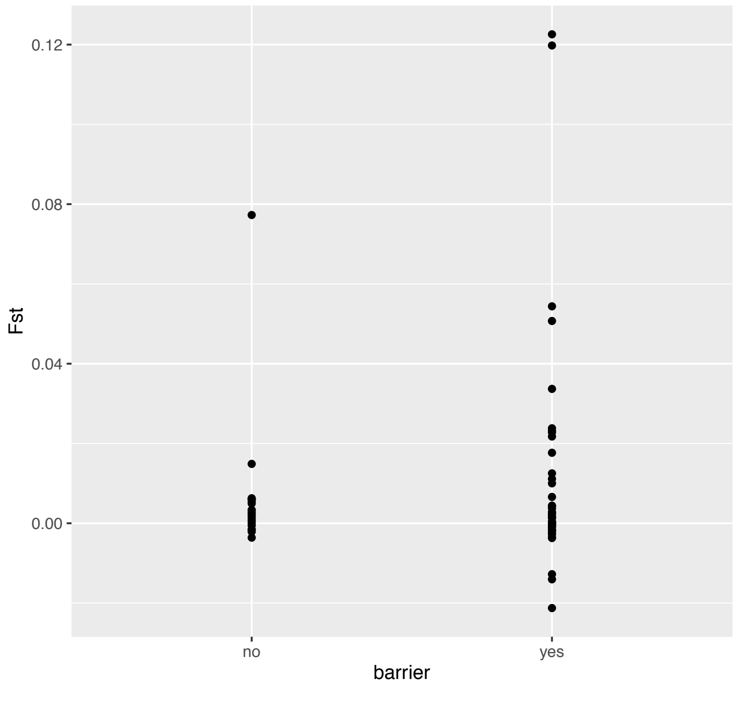

Model 2
Linear mixed model fit by REML. t-tests use Satterthwaite's method ['lmerModLmerTest']
Formula: Fst ~ QB_VR + (1 | site)
Data: Dg

REML criterion at convergence: -270.2

Scaled residuals:

Min	1Q	Median	3Q	Max
-2.3198	-0.3057	-0.1534	0.1584	3.6732

Random effects:

Groups	Name	Variance	Std.Dev.
site	(Intercept)	0.0001908	0.01381
Residual		0.0004434	0.02106

Number of obs: 64, groups: site, 15

Fixed effects:

	Estimate	Std. Error	df	t value	Pr(>|t|)
(Intercept)	0.009316	0.006624	36.85713	1.407	0.168
QB_VRPI	0.00725	0.016006	51.817602	0.453	0.652
QB_VRQB	0.002427	0.007937	55.158053	0.306	0.761
QB_VRVR	-0.006858	0.007738	57.339048	-0.886	0.379

Correlation of Fixed Effects:

	(Intr)	QB_VRP	QB_VRQ
QB_VRPI	-0.334		
QB_VRQB	-0.653	0.218	
QB_VRVR	-0.461	0.154	0.301

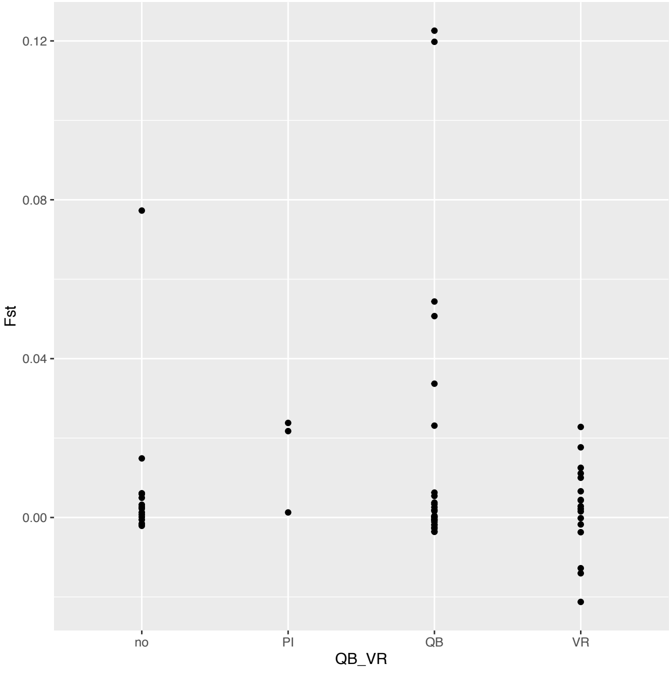

C) *Ancylus fluviatilis* (all taxa combined)

Model 1

Linear mixed model fit by REML. t-tests use Satterthwaite's method ['lmerModLmerTest']
Formula: Fst ~ barrier + (1 | site)
Data: Af

REML criterion at convergence: -334.6

Scaled residuals:

Min	1Q	Median	3Q	Max
-3.0423	-0.4379	-0.1071	0.4284	3.2007

Random effects:

Groups	Name	Variance	Std.Dev.
site	(Intercept)	0.0003382	0.01839
Residual		0.0001878	0.01371

Number of obs: 67, groups: site, 17

Fixed effects:

	Estimate	Std. Error	df	t value	Pr(>|t|)
(Intercept)	0.006888	0.005479	22.904422	1.257	0.2214
barrieryes	0.006325	0.003625	48.495829	1.745	0.0873

Correlation of Fixed Effects:

	(Intr)
barrieryes	-0.466

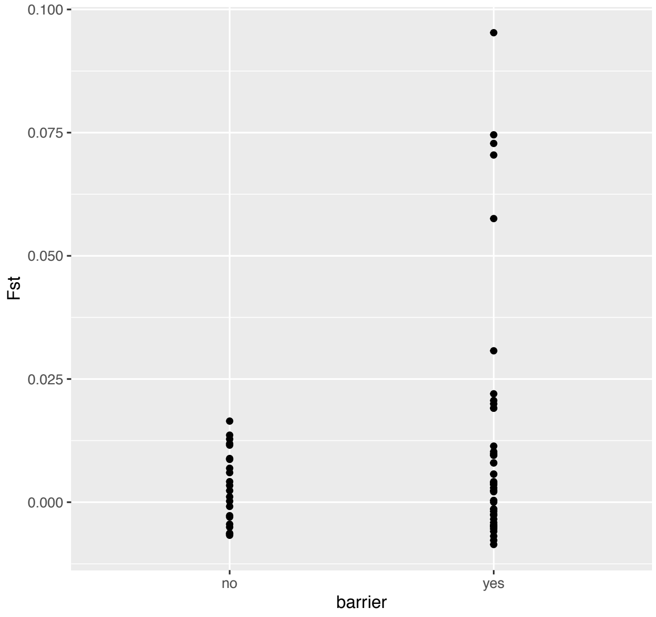

Model 2:

Linear mixed model fit by REML. t-tests use Satterthwaite's method ['lmerModLmerTest']
Formula: Fst ~ QB_VR + (1 | site)
  Data: Af

REML criterion at convergence: -345.7

Scaled residuals:

Min	1Q	Median	3Q	Max
-1.7256	-0.4464	-0.0584	0.41	3.6608

Random effects:

Groups	Name	Variance	Std.Dev.
site	(Intercept)	0.0002311	0.0152
Residual		0.0001283	0.01133

Number of obs: 67, groups: site, 17

Fixed effects:

	Estimate	Std. Error	df	t value	Pr(>|t|)
(Intercept)	7.38E-05	5.16E-03	2.55E+01	0.014	0.989
QB_VRPI	4.38E-02	7.36E-03	5.73E+01	5.95	1.71E-07
QB_VRQB	2.05E-03	4.96E-03	6.22E+01	0.414	0.68
QB_VRVR	4.97E-03	5.30E-03	5.23E+01	0.937	0.353

Correlation of Fixed Effects:

	(Intr)	QB_VRP	QB_VRQ
QB_VRPI	-0.429		
QB_VRQB	-0.532	0.228	
QB_VRVR	-0.191	0.082	0.102

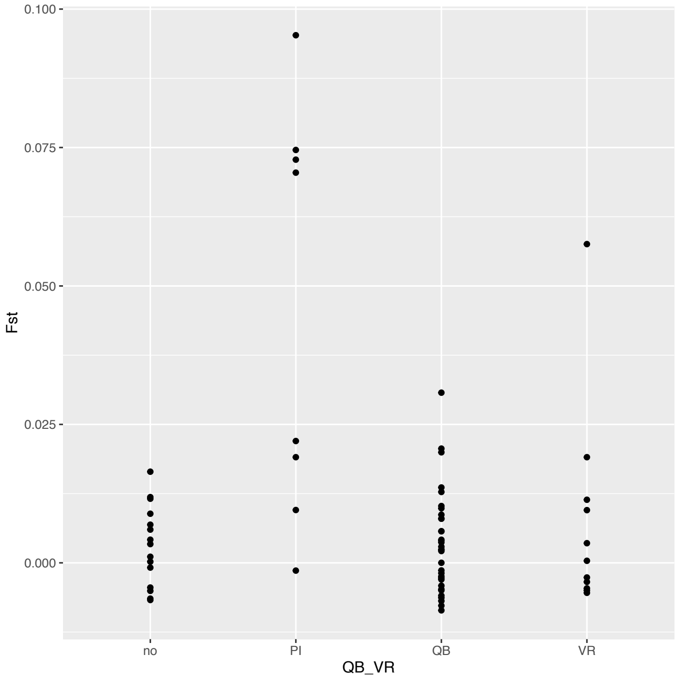

Supplement: Supplementary file 20 — Appendix S3 [file ECE3-12-e8807-s003.pdf]
